# Triple rAAV9 Vector Combinations Encoding Broadly Neutralizing Antibodies Effectively Suppress HIV-1 Infection in Humanized Mice

**DOI:** 10.3390/ijms262211051

**Published:** 2025-11-15

**Authors:** Danila S. Leontyev, Felix A. Urusov, Dina V. Glazkova, Boris V. Belugin, Anastasia A. Mitiushina, Galina M. Tsyganova, Sergey M. Yudin, Elena V. Bogoslovskaya, German A. Shipulin

**Affiliations:** Centre for Strategic Planning and Management of Biomedical Health Risks, Federal Medical Biological Agency, 119992 Moscow, Russia

**Keywords:** AAV vectors, broadly neutralizing antibodies, N6, VRC07-523, 10-1074, PGDM1400, 10E8, CombiMab-1, CombiMab-2, HIV-1, humanized mice, passive immunization, viral load, CD4^+^ T-lymphocytes

## Abstract

This study investigated the protective efficacy of two distinct combinations of three recombinant adeno-associated virus serotype 9 (rAAV9) vectors encoding broadly neutralizing antibodies against HIV-1—CombiMab-1 and CombiMab-2—in mice humanized with primary CD4^+^ T-lymphocytes. We demonstrated that mice preventively treated with CombiMab-1 or CombiMab-2 did not develop viremia and maintained human CD4+ T-lymphocyte counts following viral challenge, in contrast to control animals. These results demonstrate the significant protective capacity of CombiMab-1 and CombiMab-2 against HIV-1 challenge.

## 1. Introduction

HIV-1 infection remains one of the most significant challenges in global public health [1,2]. Despite substantial progress in the development of antiretroviral therapy (ART), which ensures effective control of viral replication and increases the life expectancy of people living with HIV-1, complete eradication of the virus remains unattainable [3,4]. According to UNAIDS, over 39 million people worldwide are currently living with HIV-1 [1], underscoring the urgent need for novel therapeutic strategies.

In recent years, therapy based on broadly neutralizing antibodies (bNAbs) has emerged as a promising approach for both prevention and treatment of HIV-1 infection [5,6]. bNAbs represent a unique class of antibodies capable of neutralizing a wide spectrum of genetically diverse HIV-1 strains, including those resistant to conventional ART [7,8]. Preclinical and clinical studies have demonstrated the substantial therapeutic potential of bNAbs to reduce viral load, control viremia after ART interruption, and, in some cases, eliminate the latent viral reservoir [9,10,11]. Approaches that enable in vivo expression of bNAbs offer new perspectives for the development of effective and long-lasting HIV treatment strategies.

A key factor in developing bNAb-based therapeutics is the rational selection of antibodies that can overcome HIV-1 diversity and provide sustained antiviral efficacy. In our previous work [12], we evaluated combinations of monoclonal antibodies targeting conserved epitopes on gp120 and gp41 that exhibited high in vitro neutralization potency against a broad spectrum of HIV-1 isolates. The use of antibody combinations targeting three distinct envelope protein (Env) epitopes represents a rational strategy to achieve potent neutralization across diverse HIV-1 strains, potentially overcoming viral immune escape mechanisms and enabling durable therapeutic responses.

Recombinant adeno-associated virus serotype 9 (rAAV9) was selected as the vector for delivering bNAbs-encoding genes. rAAV9 is distinguished by high transduction efficiency, low immunogenicity, and a well-established safety profile in both preclinical and clinical settings [13,14]. Its capacity to support sustained transgene expression makes rAAV9 a promising platform for the treatment of chronic infectious diseases, including HIV-1 [15].

As part of our ongoing work, we developed and characterized two experimental anti-HIV agents: CombiMab-1 and CombiMab-2 [16]. CombiMab-1 comprises a combination of three rAAV9 vectors encoding the N6 [17], 10-1074 [18], and 10E8 [19] antibodies, which target the CD4 binding site (CD4bs), the V3 loop, and the membrane-proximal external region (MPER), respectively. CombiMab-2 consists of three rAAV9s encoding VRC07-523 [20], PGDM1400 [21], and 10-1074, directed against CD4bs, V1/V2 region, and V3 loop epitopes (Table 1). In our previous studies using C57BL/6 mice, rAAV administration resulted in sustained antibody expression and broad, potent HIV-1-neutralizing activity in serum.

The aim of this study was to assess the protective efficacy of CombiMab-1 and CombiMab-2 against HIV-1 infection using a humanized NOD-*Prkdc*^scid^*Il2r*g^em1^/^Smoc^ (NMG) mouse model.

## 2. Results

### 2.1. Serum Levels of Broadly Neutralizing Antibodies Following Administration of CombiMab-1 and CombiMab-2

To evaluate the efficiency of rAAV9-mediated delivery and expression of bNAbs, CombiMab-1 and CombiMab-2 were administered concurrently to two groups of mice. Forty-six days post-injection, human IgG plasma concentrations were quantified.

**Figure 1:**
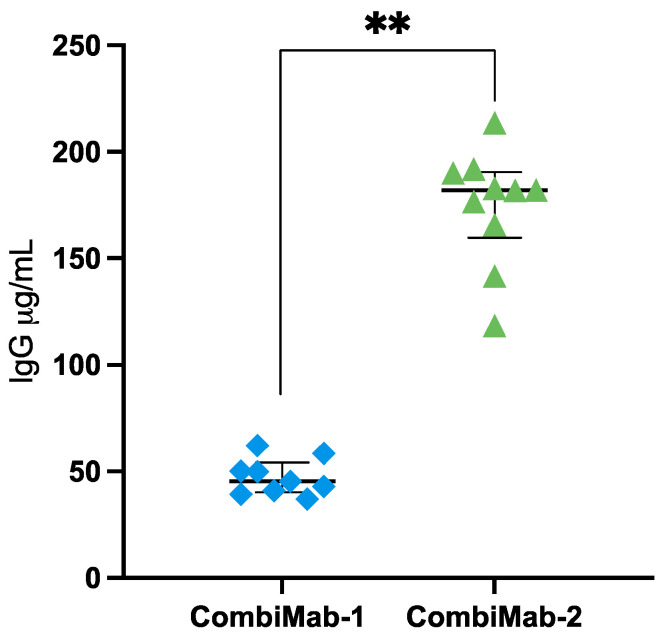
Human IgG concentrations in the plasma of NMG mice 46 days after immunization with CombiMab-1 (n = 9) and CombiMab-2 (n = 10). Individual values (µg/mL plasma), median concentrations (horizontal line), and IQR are shown. Statistical significance was assessed using the Mann–Whitney U test. ** *p* = 0.000022.

In the CombiMab-1 group, the median serum IgG concentration was 45.4 µg/mL (interquartile range [IQR], 40–54 µg/mL) (Figure 1, Table S1). In contrast, mice receiving CombiMab-2 exhibited significantly higher IgG levels, with a median of 182 µg/mL (IQR, 159–190 µg/mL).

### 2.2. Protective Efficacy of CombiMab-1

**Figure 2:**
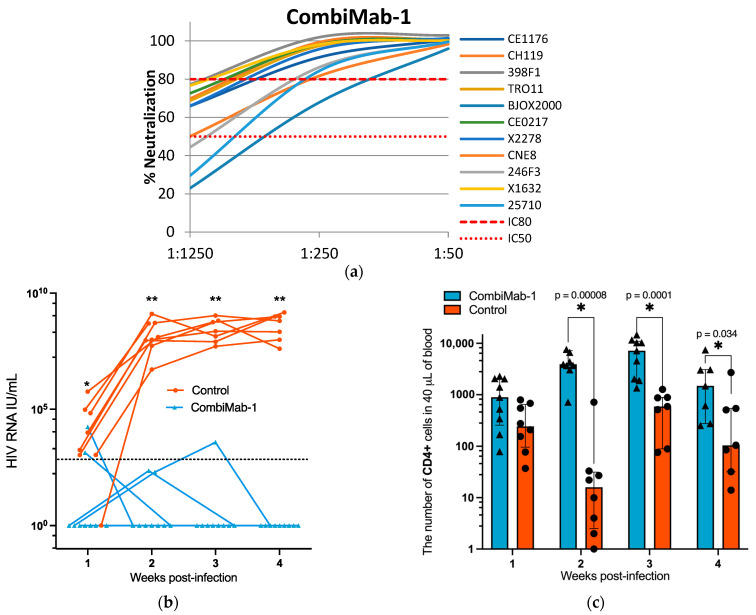
Protective efficacy of CombiMab-1 against HIV-1 infection in humanized mice. (**a**) Neutralizing activity of pooled sera from NMG mice treated with CombiMab-1 against a global panel of HIV-1 pseudoviruses. (**b**) Plasma HIV-1 RNA levels (IU/mL) in infected mice. Solid lines connect individual viral load values across time points for each mouse. The dashed horizontal line indicates the lower limit of quantification (LLOQ) for the RT-PCR assay (860 IU/mL). (**c**) Absolute counts of hCD4^+^ T cells in the peripheral blood of CombiMab-1-treated and control mice. Dots represent individual values; bars indicate medians; whiskers denote interquartile ranges. Statistical significance was determined using the Mann–Whitney U test. * *p* < 0.05, ** *p* < 0.01.

The neutralizing activity of pooled sera from CombiMab-1-treated NMG mice was assessed against a global panel of HIV-1 pseudoviruses, as shown in Figure 2a. At a serum dilution of 1:250, neutralization efficiency ranged from 80 to 100% for most pseudoviruses except the BJO2000 strain. At the lowest tested dilution (1:50), the pooled sera effectively neutralized all pseudoviruses within the global HIV-1 panel (Figure 2a).

To evaluate the in vivo protective efficacy of CombiMab-1, we utilized a humanized mouse model established by the transfer of human CD4^+^ (hCD4^+^) T cells into immunodeficient NMG mice.

The timelines of the two independent experiments using CombiMab-1 and CombiMab-2 are summarized in Figure S1. Each timeline indicates the key experimental procedures, including the administration of rAAV9 vectors and subsequent HIV-1 challenge, allowing a clear comparison between the two study designs.

We have previously demonstrated the suitability of this model for studying anti-HIV therapeutics [22]. A cohort of mice (n = 9) was humanized with hCD4^+^ T cells 46 days after administration of CombiMab-1, alongside a control group (n = 8). All animals were challenged with HIV-1 one day after humanization.

Plasma viral RNA levels were measured weekly post-infection. All animals in the control group developed viremia, with a median viral load of 5.9 × 10^3^ IU/mL at one week post-infection, which increased to 6.5 × 10^8^ IU/mL by week four (Figure 2b, Table S2). In contrast, among the CombiMab-1-treated mice, five maintained undetectable levels of HIV-1 RNA throughout the observation period. Three mice exhibited sporadic single-point low-level viremia (Table S2), and one animal displayed a transient, modest viral load at weeks two and three, which declined to undetectable levels thereafter.

**Figure 3:**
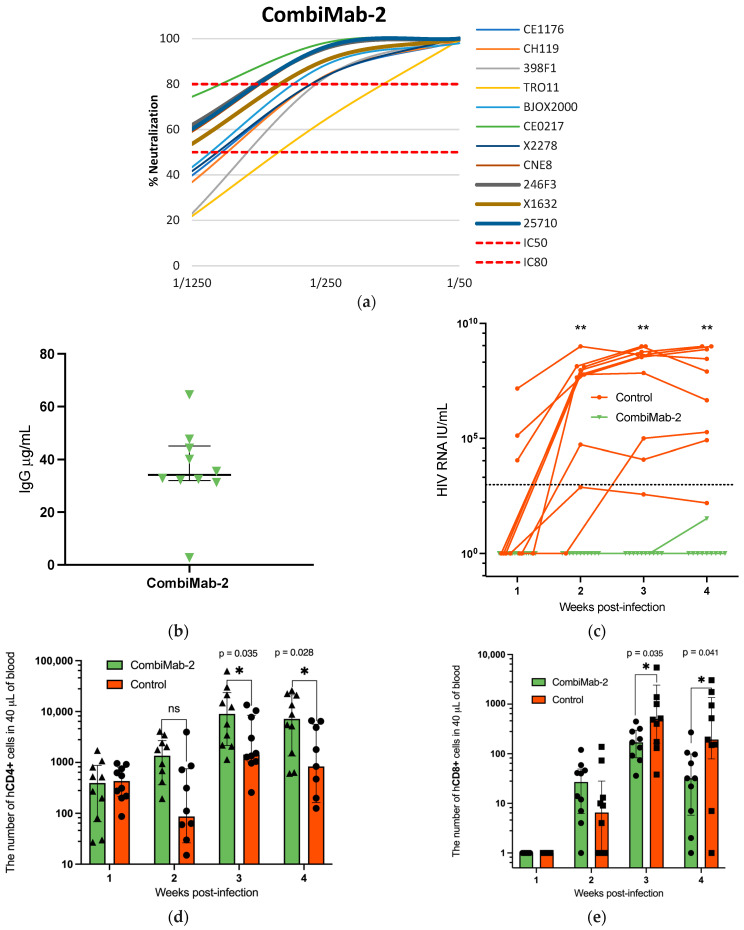
Protective effect of CombiMab-2 against HIV infection in humanized mice. (**a**) Neutralizing activity of pooled sera from NMG mice treated with CombiMab-2 against the global panel of HIV-1 pseudoviruses. (**b**) Concentration of human IgG in mouse plasma 28 days after CombiMab-2 administration. Data points represent individual values (µg/mL); horizontal lines indicate median value; and whiskers represent IQR, n = 10. (**c**) Plasma HIV-1 RNA levels in individual mice over time. Solid lines connect viral load values for each mouse; the black dashed line denotes the LLOQ for the PCR assay (860 IU/mL). Absolute counts of hCD4^+^ (**d**) and hCD8^+^ T cells (**e**) T cells in the peripheral blood of CombiMab-2-treated and control mice. Data points represent individual values; bars show medians, and whiskers indicate the IQR. Statistically significant differences between groups are denoted as *—*p* < 0.05, **—*p* < 0.01 (Mann–Whitney U-test).

To monitor changes in the hCD4^+^ T cell population during the course of the study, peripheral blood samples were analyzed by flow cytometry. From the second week post-infection onward, the number of circulating hCD4^+^ T cells was significantly higher in CombiMab-1-treated mice compared to controls (*p* < 0.05, Mann–Whitney U test; Figure 3b).

### 2.3. Protective Efficacy of CombiMab-2

A separate experiment was conducted to evaluate the protective efficacy of CombiMab-2. One group of NMG mice received CombiMab-2, while a second group served as untreated controls. At 28 days post-administration, the median concentration of human IgG in serum was 34 µg/mL (IQR 32–45 µg/mL) (Figure 3b, Table S1).

As in the previous experiment, the neutralization activity of pooled sera from treated mice was assessed against a global panel of HIV-1 pseudoviruses. At a 1:250 dilution, the pooled sera neutralized all tested pseudoviruses with ≥80% efficiency, with the exception of TRO11, which was neutralized at a 64% rate (Figure 3a). Notably, at a 1:50 dilution, the pooled sera completely neutralized all viruses in the panel, including TRO11, achieving 100% inhibition.

Twenty-eight days after CombiMab-2 administration, mice were humanized with hCD4^+^ T cells and challenged with HIV-1 the following day, using the same protocol as in the CombiMab-1 study. All control mice developed detectable viremia, with only one of ten showing plasma viral RNA levels below the lower limit of quantification (LLOQ) of the PCR assay. Plasma viral load kinetics are shown in Figure 3c and detailed in Table S3. In the CombiMab-2–treated group, HIV-1 RNA remained undetectable throughout the study, with the exception of a single mouse that exhibited a transient, low-level signal below the LLOQ at week 4.

Flow cytometric analysis of the hCD4^+^ cell population showed that during the first week post-infection, cell counts in the CombiMab-2 group were comparable to those in the control group. By the second week, a trend toward increased hCD4^+^ cell numbers was observed in Combimab-2-treated mice, although these differences did not reach statistical significance. By weeks three and four, hCD4^+^ T cell counts were significantly higher in the Combimab-2 group compared to the controls (Figure 3d). A similar temporal pattern and intergroup differences were also observed in the CombiMab-1 study.

In the first experiment, despite administering a purified hCD4^+^ cell fraction to the mice, flow cytometric analysis at the 4th week after T-cell transplantation revealed the presence of CD3^+^CD4^−^ cells (Figure S2), likely representing CD3^+^CD8^+^ T lymphocytes. Therefore, in the second experiment, we monitored human CD8^+^ (hCD8^+^) T cells’ dynamics in peripheral blood. hCD8^+^ T cells were undetectable in all groups of mice during the first week. They began to appear by week 2, with progressive increases observed through weeks 3 and 4 (Figure 3e). Notably, hCD8^+^ T cell counts at post-infection weeks 3 and 4 were significantly lower in the CombiMab-2 group compared to controls (*p* < 0.05, Mann–Whitney U-test).

## 3. Discussion

In this study, we evaluated the antiviral efficacy of two rAAV9 vector combinations—CombiMab-1 and CombiMab-2—in a humanized mouse model of HIV-1 infection. Consistent with previous results in C57BL/6 mice [16], CombiMab-2 induced higher serum levels of bNAbs compared to CombiMab-1 in the first experiment. Notably, in the second experiment, the antibody concentrations in the CombiMab-2 group were lower than those observed in the first experiment, likely due to the earlier sampling time point (28 days vs. 46 days after administration). This difference may reflect continued accumulation of circulating bNAbs between weeks 4 and 6 after rAAV9 administration. A similar upward trend in antibody titers over this interval has been reported previously in C57BL/6 mice [16] and in immunodeficient Rag2 ^−/−^γc ^−/−^ mice [23]. Nevertheless, variability between experiments may also reflect differences in rAAV9 production batches, animal cohorts, or sampling schedules.

Neutralization assays performed with sera from NMG mice treated with CombiMab-1 or CombiMab-2 produced results consistent with previous observations in C57BL/6 mice. Both treatment groups exhibited potent neutralizing activity against the majority of pseudoviruses included in the global panel of HIV-1 pseudoviruses. As reported previously, sera from the CombiMab-1 group demonstrated the weakest neutralizing activity against the BJOX2000 pseudovirus. Importantly, the overall neutralizing potency of CombiMab-1 sera was greater in NMG mice compared to C57BL/6 mice, with a 1:250 serum dilution achieving over 50% neutralization across all tested pseudoviruses, in contrast to the 1:25 dilution required in C57BL/6 mice. This enhanced potency likely reflects higher circulating antibody concentrations in NMG mice. In contrast, the neutralizing activity of CombiMab-2 sera was comparable between the two mouse models, with the lowest activity observed against the TRO11 pseudovirus, consistent with prior data [16].

In HIV-1 challenge experiments, all animals in the control group developed sustained viremia, confirming the suitability and consistency of the model for evaluating the in vivo efficacy of therapeutic candidates. Both CombiMab-1 and CombiMab-2 effectively prevented the progression of viremia in treated animals. Importantly, no statistically significant differences in antiviral efficacy were observed between CombiMab-1 and CombiMab-2 in this study. In the CombiMab-1 evaluation experiment, low-level viremia was transiently detected in some mice at early time points, followed by a decline below the detection threshold without evidence of subsequent viral rebound. The transient spike in plasma viral RNA observed in these animals may reflect the relatively high viral challenge dose used in this study. This may explain why complete protection was not achieved in all CombiMab-1–treated mice. In contrast, complete suppression of viral replication was observed in all animals treated with CombiMab-2 throughout the second experiment. Notably, viremia in control mice was more pronounced in the first experiment than in the second, potentially reflecting differences in mouse cohorts or partial degradation of the viral inoculum. The more robust infection observed in the first experiment likely imposed more stringent conditions for therapeutic assessment, possibly accounting for the transient viremia observed in the CombiMab-1 group. Importantly, these transient episodes did not progress to persistent infection. Although differences in infection dynamics between experiments limit direct comparisons of CombiMab-1 and CombiMab-2, both therapeutics demonstrated broadly similar neutralizing activity against viruses from the global HIV-1 panel. Meaningful differences in therapeutic efficacy, if any, may only become apparent in future studies using additional challenge strains or clinical isolates.

Another indicator of the protective efficacy of CombiMab-1 and CombiMab-2 was the higher number of hCD4⁺ lymphocytes observed in AAV-treated animals compared with controls at 2, 3, and 4 weeks post-infection. The control group exhibited a clear decline in hCD4⁺ lymphocyte numbers by the second week post-infection. Although partial recovery was noted thereafter, CD4⁺ T cell levels remained significantly lower than in either treatment group throughout the study. The reduction in hCD4⁺ counts in controls can be attributed to the cytopathic effects of HIV-1. Consistent with this, previous studies by Balazs et al., using a comparable PBMCs-humanized mouse model, demonstrated that HIV-1 typically induces a marked decline in hCD4⁺ lymphocyte frequencies within 2–4 weeks post-infection, depending on the viral dose [23,24]. Thus, sustained preservation of hCD4⁺ lymphocytes in CombiMab-treated animals is consistent with a protective therapeutic effect.

In the second experiment, we additionally monitored hCD8⁺ T-lymphocyte dynamics, because proliferation of this subpopulation may serve as a marker of the graft-versus-host disease (GvHD) starting. Previous studies have also linked the expansion of hCD8^+^ T cells in humanized mice to the development of GvHD [25,26,27,28]. In our experiments, the increase in hCD8^+^ T cell numbers was accompanied by a statistically significant decline in body weight, consistent with the onset of GvHD, with a trend toward more pronounced weight loss in the control group (Tables S4 and S5).

One possible explanation for the principal emergence of hCD8^+^ T cells following the transplantation of in vitro expanded hCD4^+^ cells is the presence of residual hCD8^+^ T cells among the purified hCD4^+^ T-lymphocyte fraction, resulting from an imperfect purification procedure, as supported by our flow cytometry data (Figure S3). Additionally, some studies have demonstrated phenotypic plasticity in CD4⁺ T cells, wherein strong activation can induce CD8 expression and the acquisition of cytotoxic functions [29]. Evidence of in vivo lineage conversion of murine lymphocytes under conditions of immune activation [30] further supports the possibility that similar mechanisms may contribute to the generation of hCD8^+^ T cells within our chimeric immune system. We did not exclude the contribution of HIV-1 replication to hCD8^+^ cells proliferation rate: despite the expansion of this population being observed in both the control and CombiMab-2 groups, significantly greater counts of hCD8^+^ T cells were observed in the control group with high viral load. However, the precise mechanisms underlying the appearance and differential expansion of hCD8^+^ T cells in animals with high viral loads remain unclear and require further investigation.

The observed antiviral efficacy of CombiMab-1 and CombiMab-2 is consistent with prior studies utilizing AAV vectors for the bNAbs expression in humanized mouse models of HIV-1, both for prophylaxis and therapeutic intervention. For example, Balazs et al., 2011, reported that rAAV-mediated expression of the VRC01 antibody effectively suppressed plasma viremia in NSG mice [23]. Similarly, Horwitz et al., 2013, demonstrated that in vivo expression of the 10-1074 antibody conferred durable protection against HIV-1 for over 60 days in a comparable model [31]. A distinguishing feature of our study is the use of triple combinations of bNAbs that target distinct viral epitopes (Table 1), a strategy that may reduce the likelihood of viral escape by simultaneously limiting multiple evolutionary pathways to resistance. This combinatorial approach is expected to offer enhanced protection compared to monotherapy, as previously noted by Caskey 2020 [32].

Despite the technical simplicity and reproducibility of the transient humanization strategy employed in this study, based on the purified hCD4^+^ T cell transfer, it did not prevent the development of GvHD, in contrast to findings reported by Søndergaard et al., 2013 [33]. The onset of GvHD markedly restricted the duration of post-challenge monitoring and limited our ability to assess the long-term stability of the protective effect, representing a significant limitation of the current study.

Nevertheless, our in vivo data demonstrate that both CombiMab-1 and CombiMab-2 support sustained expression of bNAbs, suppress HIV-1 replication, and preserve hCD4^+^ T cell populations over the course of infection. These readouts are widely accepted as critical preclinical markers of anti-HIV efficacy and provide a strong translational bridge to human clinical application [6,7,34,35]. Future investigations could further validate the efficacy of CombiMab-1 and CombiMab-2 using advanced humanized mouse models, such as CD34⁺-reconstituted NSG or BLT mice, which support more complete human immune system reconstitution [36,37,38,39]. These models would permit the evaluation of long-term therapeutic durability, potential viral escape, and efficacy in the context of established HIV-1 infection.

Although the humanized model used in our study has inherent constraints—such as incomplete immune reconstitution and restricted observation time—our findings provide important translational insights that directly inform the design of next-generation anti-HIV therapies based on recombinant AAV delivery of bNAbs genes [40,41].

In summary, these findings underscore the promise of the CombiMab platform as a viable strategy for HIV-1 prophylaxis and treatment and provide strong justification for further preclinical development and eventual human testing.

## 4. Materials and Methods

The study was conducted in accordance with the Declaration of Helsinki, and the protocol was approved by the Ethics Committee of the “CSP” FMBA of Russia (protocol no. 2) on 15 February 2024.

### 4.1. Animals

Female and male immunodeficient NOD-*Prkdc*^scid^*Il2r*g^em1/Smoc^ (NMG) mice, aged 6–8 weeks, were obtained from the animal facility of the Shemyakin-Ovchinnikov Institute of Bioorganic Chemistry (Pushchino, Russia). Animals were housed in individually ventilated cages (ISOCage, Tecniplast, Buguggiate, Italy) under specific pathogen-free (SPF) conditions. Body weight was monitored weekly. All procedures involving HIV-1 and infected animals were conducted under biosafety level 3 conditions.

### 4.2. Production of CombiMab-1 and CombiMab-2

Production of rAAV9 vectors for CombiMab-1 and CombiMab-2 was performed as previously described [16]. HEK293FT cells were transfected with three plasmids: *pAAV-Helper* (Cell Biolabs, San Diego, CA, USA), *pAAV-RC-9* (Cell Biolabs, USA), and a vector plasmid encoding the respective antibody, using PEI MAX 40000 (Polysciences, Warrington, PA, USA).

Seventy-two hours post-transfection, cells were harvested, lysed, and treated with benzonase (Merck Life Science, Darmstadt, Germany). rAAV9 vectors were purified by iodixanol density gradient ultracentrifugation (Unique Pharmaceuticals Laboratories, Prabhadevi, Mumbai, India), followed by dialysis and concentration using Amicon Ultra-50K filters (Merck Life Science, Rahway, NJ, USA). Final rAAV9 vector preparations were aliquoted and stored at −70 °C.

Vector genome (vg) copies in rAAV9 samples were quantified by droplet digital PCR (ddPCR) using the AmpliTest RIBO-prep RNA/DNA extraction kit (CSP FMBA, Moscow, Russia), ddPCR™ reagents and TaqMan QX100/QX200™ probes, and a QX200™ AutoDG™ and C1000 Touch Thermal Cycler system (Bio-Rad, Hercules, CA, USA). The primers used were as follows: forward, 5′-CCTTGTATAAATCCTGGTTGCTGTCT-3′; reverse, 5′-GGAAAGGAGCTGACAGGTGGT-3′; and probe, R6G–TCAGGCAACGTGGCGTGGTGTG–BHQ1 (synthesized by GenTerra, Moscow, Russia), targeting the *WPRE* sequence in rAAV constructs.

### 4.3. Passive Immunization

Twenty-nine to forty-seven days prior to HIV-1 infection, mice in the experimental groups received one of the rAAV9 combinations, CombiMab-1 or CombiMab-2. Each of the three rAAV9 vectors comprising the formulations was administered separately into different muscles at a dose of 4 × 10^11^ vg per mouse. Control animals received an equivalent volume of 0.9% NaCl solution. To confirm expression of broadly neutralizing antibodies at 28 or 46 days post-administration, mouse blood samples were collected on day −1; sera were isolated and heat-inactivated at 56 °C for 30 min, and human IgG concentrations were quantified using a commercial Human IgG ELISA Antibody Pair Kit (StemCell, Vancouver, BC, Canada), according to the manufacturer’s instructions.

### 4.4. Humanized Mouse Model

Whole blood leukoconcentrate was obtained from a healthy donor with informed consent. On the same day, human peripheral blood mononuclear cells (PBMCs) were isolated by density gradient centrifugation using Ficoll solution (P050E, PanEco, Moscow, Russia). Human CD4^+^ (hCD4^+^) T-lymphocytes were then isolated from PBMCs by negative magnetic selection using the CD4+ T Cell Isolation Kit (Cat. No. 130-096-533, Miltenyi Biotec, Bergisch Gladbach, Germany). The purity of isolated cells was verified by antibody staining and flow cytometry; only samples containing ≥95% CD4^+^ T cells were cryopreserved and stored for further use. Ten days before humanization, cells were thawed and resuspended in serum-free X-VIVO 15 medium (Lonza, Basel, Switzerland) at a density of 2 × 10^6^ cells/mL (day −10). From this point onward, cells were cultured at 37 °C in a humidified atmosphere with 5% CO_2_. On the following day (day −9), cells were diluted to 1 × 10^6^ cells/mL and activated using CD3/CD28 Dynabeads Human T-Activator magnetic particles (Gibco, Thermo Fisher Scientific, Waltham, MA, USA). Interleukin-2 (IL-2, Biotech LLC, Moscow, Russia) was added to a final concentration of 100 IU/mL. Cells were subsequently cultured in X-VIVO 15 medium supplemented with 100 IU/mL IL-2. On day −6, magnetic particles were removed, and the cell concentration was adjusted to 1 × 10^6^ cells/mL. On days −5 and day −3, cell concentrations were adjusted to 0.5 × 10^6^ cells/mL and 1 × 10^6^ cells/mL, respectively. On day −1, cell viability was assessed using 0.1% trypan blue. Cells were centrifuged at 300× *g* for 5 min, and the resulting pellet was resuspended in phosphate-buffered saline (DPBS, Gibco, Waltham, MA, USA) at a final concentration of 7.5 × 10^7^ cells/mL. On the same day, humanization was performed by intravenous injection of 1.5 × 10^7^ hCD4^+^ T cells in 200 μL per mouse via the tail vein. Two weeks post-humanization, mice received an intraperitoneal injection of normal human immunoglobulin (Microgen JSC, Moscow, Russia) at a dose of 2 g/kg to transiently suppress graft-versus-host disease (GvHD).

### 4.5. HIV-1 Production

Human embryonic kidney cells HEK-293FT (Thermo Fisher Scientific Inc., Waltham, MA, USA), cultured in Dulbecco’s Modified Eagle Medium (DMEM) supplemented with 10% fetal bovine serum (FBS; HyClone, Marlborough, MA, USA), 1% MEM NEAA 100× (Gibco), 10 mM 4-(2-hydroxyethyl)-1-piperazineethanesulfonic acid (HEPES), and streptomycin/penicillin at 100 μg/mL and 100 IU/mL, respectively (Gibco), at 37 °C with 5% CO_2_ were used to produce HIV-1. The CCR5-tropic HIV-1 strain NL4-3(AD8) was produced by transfecting HEK-293FT cells with the *pNL4-3(AD8)* plasmid (provided by the NIH AIDS Research and Reference Reagent Program). Transfection was performed using FuGENE (Promega, Madison, WI, USA). Sixteen hours after transfection, the medium was replaced with serum-free OptiMEM (Thermo Fisher Scientific, Waltham, MA, USA). On day 3 post-transfection, the cell supernatant was collected, filtered through a 0.45 µm filter (TPP, Trasadingen, Switzerland), treated with benzonase, and concentrated 10-fold using Amicon^®^ Ultra-15 centrifugal filters (Merck Life Science, Rahway, NJ, USA) with a 100 kDa cutoff. The viral suspension was aliquoted and stored at −70 °C. The concentration of HIV-1 p24 antigen in the viral suspension was measured using the HIV-1 p24 ELISA kit (Ecolab JSC, Moscow, Russia) according to the manufacturer’s instructions.

### 4.6. HIV-1 Infection and Blood Sampling

Mice were infected one day after administration of hCD4^+^ T cells. Both experimental and control groups were challenged with HIV-1 NL4-3(AD8) at a dose of 500 ng p24 per mouse via intraperitoneal injection. Blood was collected weekly to monitor viral load and T-cell counts, via retro-orbital or femoral vein puncture under isoflurane anesthesia. Blood was collected using capillary tubes Microvette 200 (Sarstedt, Nümbrecht, Germany), with 20 μL of 0.5 M ethylenediaminetetraacetic acid (EDTA) added to prevent coagulation.

### 4.7. Assessment of Viral Load in Mouse Plasma Samples

Blood samples were centrifuged at 1000× *g* for 10 min to separate plasma. The cell pellet was retained for cytometric analysis. HIV-1 RNA levels in mouse plasma were quantified by reverse transcription-polymerase chain reaction (RT-PCR) using the RealBest HIV RNA Quantitative kit (Vector-Best JSC, Koltsovo, Novosibirsk Oblast, Russia) according to the manufacturer’s instructions.

### 4.8. Flow Cytometry Analysis

Cell pellets obtained after centrifugation were resuspended in phosphate-buffered saline to the original plasma volume, and 40 µL was used for staining with fluorophore-conjugated antibodies specific for human receptors: hCD45-APC, hCD3-FITC, hCD4-APC-Cy7, hCD8-BV421 (BioLegend, San Diego, CA, USA). Flow cytometric analysis and quantification of human T lymphocytes were performed using the Bio-Rad ZE5 flow cytometer (Bio-Rad, Hercules, CA, USA).

### 4.9. Neutralization Assay

Neutralization assays were performed using TZM-bl cells (NIH AIDS Reagent Program, Manassas, VA, USA), cultured in DMEM supplemented with 10% FBS and 25 mM HEPES. HIV-1 pseudoviruses from the global panel (cat# 12670, NIH AIDS Reagent Program, Germantown, MD, USA) were produced and titrated as described in Shipulin et al., 2024 [16]. Five-fold serial dilutions of mouse serum were used to assess neutralizing activity following the same protocol.

### 4.10. Statistical Analysis

Data were analyzed using GraphPad Prism 9 (GraphPad Software, San Diego, CA, USA). The Mann–Whitney nonparametric test was employed to assess statistical differences between groups. A significance level of *p* ≤ 0.05 was considered statistically significant.

## Figures and Tables

**Table 1:** Targets and encoded antibodies in CombiMab-1 and CombiMab-2 rAAV9 vectors.

Target	CombiMab-1: rAAV9 Vectors Encoding Antibodies	CombiMab-2: rAAV9 Vectors Encoding Antibodies
CD4bs	N6	VRC07-523
V1/V2	-	PGDM1400
V3	10-1074	10-1074
MPER	10E8	-

## Data Availability

The original contributions presented in this study are included in the article/Supplementary Materials. Neutralization assay and flow cytometry data available on request from the authors.

## Supplementary Materials

The following supporting information can be downloaded at: https://www.mdpi.com/article/10.3390/ijms262211051/s1.

## Abbreviations

The following abbreviations are used in this manuscript:

rAAV9	recombinant adeno-associated virus serotype 9
HIV	human immunodeficiency virus
ART	antiretroviral therapy
bNAbs	broadly neutralizing antibodies
CD4bs	CD4 binding site
IQR	interquartile range
MPER	membrane-proximal external region
PBMCs	human peripheral blood mononuclear cells
GvHD	graft-versus-host disease
LLOQ	lower limit of quantification
