# Supplementary material for: Triple rAAV9 Vector Combinations Encoding Broadly Neutralizing Antibodies Effectively Suppress HIV-1 Infection in Humanized Mice
Source: Int J Mol Sci. 2025 Nov 15;26(22):11051. doi: 10.3390/ijms262211051 (PMC12652006; doi:10.3390/ijms262211051)
Supplement: Supplementary file 1 [file ijms-26-11051-s001.zip › ijms-3917548-supplementary.pdf]

SUPPLEMENTARY

Table S1 Plasma concentrations of human IgG in NMG humanized mice before HIV-1 infection. Data from two independent experiments (Experiment 1 and Experiment 2) are shown. Values for individual mice are presented along with the median and interquartile range (IQR)

Experiment 1				Experiment 2	
**Animal №**	**CombiMab-1**	**Animal №**	**CombiMab-2**	**Animal №**	**CombiMab-2**
1.1	62.1 µg/mL	3.4	213.5 µg/mL	1.1	31.3 µg/mL
1.2	42.9 µg/mL	4.1	190 µg/mL	1.2	44.2 µg/mL
1.3	39.3 µg/mL	4.2	165.7 µg/mL	1.3	32.2 µg/mL
1.4	37 µg/mL	4.3	183 µg/mL	2.1	32.5 µg/mL
1.5	50.1 µg/mL	4.4	176.6 µg/mL	2.2	64.5 µg/mL
2.1	58.4 µg/mL	4.5	182 µg/mL	2.3	2.7 µg/mL
5.1	45.4 µg/mL	6.1	141.7 µg/mL	3.1	32.9 µg/mL
5.2	41.0 µg/mL	6.2	118.3 µg/mL	3.2	40.1 µg/mL
5.3	49.8 µg/mL	6.3	191.9 µg/mL	3.3	35.5 µg/mL
-	-	6.4	181.9 µg/mL	3.4	47.7 µg/mL
**Median (Q1-Q3)**	**45.4 (40.15-54.25)**	**Median (Q1-Q3)**	**182.0 (159.7-190.5)**	**Median (Q1-Q3)**	**34.2 (31.9-45.1)**

Table S2 Plasma viral RNA concentrations in infected mice. Median values are shown for Experiment 1

	Animal №	HIV-1 RNA. IU/mL			
		**Week 1**	**Week 2**	**Week 3**	**Week 4**
**Control**	2.2	9.7E+04	5.0E+08	-	-
	2.3	6.9E+04	1.3E+09	1.4E+08	1.5E+09
	3.2	8.6E+02	5.3E+08	1.1E+09	6.5E+08
	7.1	0.0+00	5.5E+07	6.6E+08	4.1E+07
	7.2	1.1E+03	1.0E+08	2.3E+08	2.2E+08
	8.1	1.1E+03	5.2E+06	5.2E+07	1.0E+08
	8.2	5.8E+05	1.3E+08	5.7E+08	1.0E+09
	8.3	1.0E+04	9.3E+07	8.2E+07	1.0E+09
**CombiMab-1**	1.1	0.0+00	2.3E+02	0.0+00	-
	1.2	1.4E+03	0.0+00	0.0+00	0.0+00
	1.3	0.0+00	0.0+00	0.0+00	0.0+00
	1.4	0.0+00	0.0+00	0.0+00	0.0+00
	1.5	0.0+00	0.0+00	0.0+00	0.0+00
	2.1	0.0+00	0.0+00	0.0+00	0.0+00
	5.1	0.0+00	2.0E+02	3.8E+03	0.0+00
	5.2	0.0+00	0.0+00	0.0+00	0.0+00
	5.3	1.8E+04	0.0+00	0.0+00	0.0+00
**Median (Q1-Q3). Control**		**5.9E+03 (1.1E+03-9.00E+04)**	**1.15E+08 (6.45E+07-5.23E+08)**	**2.30E+08 (8.20E+07-6.60E+08)**	**6.50E+08 (1.00E+08-1.00E+09)**
**Median (Q1-Q3). CombiMab-1**		**0.0E+00 (0.0E+00-7.0E+02)**	**0.0E+00 (0.0E+00-1.0E+02)**	**0.0E+00 (0.0E+00-0.0E+00)**	**0.0E+00 (0.0E+00-0.0E+00)**
**Mann-Whitney test. p-value**		**0.011436**	**0.000082**	**0.000087**	**0.000087**

Table S3 Plasma viral RNA concentrations in infected mice. Median values are shown for Experiment 2

	Animal №	HIV-1 RNA. IU/mL			
		**Week 1**	**Week 2**	**Week 3**	**Week 4**
**Control**	4.1	1.5E+07	1.0E+09	4.5E+08	2.9E+08
	4.2	0.0+00	5.4E+04	1.2E+04	8.3E+04
	4.3	0.0+00	9.1E+07	5.6E+08	1.0E+09
	4.4	0.0+00	7.6E+02	3.7E+02	1.6E+02
	4.5	0.0+00	5.9E+07	4.7E+08	1.0E+09
	5.1	0.0+00	4.4E+07	3.4E+08	7.4E+08
	5.2	0.0+00	1.3E+08	1.0E+09	-
	5.3	1.1E+04	1.4E+08	1.0E+09	8.0E+07
	5.4	0.0+00	0.0+00	9.9E+04	1.9E+05
	5.5	1.3E+05	6.0E+07	6.9E+07	4.5E+06
**CombiMab-2**	1.1	0.0+00	0.0+00	0.0+00	0.0+00
	1.2	0.0+00	0.0+00	0.0+00	0.0+00
	1.3	0.0+00	0.0+00	0.0+00	0.0+00
	2.1	0.0+00	0.0+00	0.0+00	0.0+00
	2.2	0.0+00	0.0+00	0.0+00	0.0+00
	2.3	0.0+00	0.0+00	0.0+00	0.0+00
	3.1	0.0+00	0.0+00	0.0+00	0.0+00
	3.2	0.0+00	0.0+00	0.0+00	0.0+00
	3.3	0.0+00	0.0+00	0.0+00	3.3E+01
	3.4	0.0+00	0.0+00	0.0+00	0.0+00
**Median (Q1-Q3). Control**		**0.00E+00 (0.00E+00-4.08E+04)**	**5.95E+07 (4.07E+04-1.33E+08)**	**3.95E+08 (7.73E+04-6.70E+08)**	**8.00E+07 (1.37E+05-8.70E+08)**
**Median (Q1-Q3). CombiMab-2**		**0.0E+00 (0.0E+00-0.0E+00)**	**0.0E+00 (0.0E+00-0.0E+00)**	**0.0E+00 (0.0E+00-0.0E+00)**	**0.0E+00 (0.0E+00-0.0E+00)**
**Mann-Whitney test. p-value**		**0.210526**	**0.000119**	**0.000011**	**0.000022**

Table S4 Mean body weight of animals during Experiment 1

	Mean body weight (g). ±SEM				Weight decrease from previous time point (%)		
	Week 1	Week 2	Week 3	Week 4	Week 2	Week 3	Week 4
**CombiMab-1**	22.40 ± 0.82	20.60 ± 0.43	17.80 ± 0.30	15.10 ± 0.32	7.4%	20.6%	32.7%
**Control**	25.20 ± 0.59	22.30 ± 0.69	19.60 ± 0.56	16.20 ± 0.60	10.9%	20.8%	34.4%
**T test**	0.014	0.065	0.074	0.162	0.4	0.963	0.67

Table S5 Mean body weight of animals during Experiment 2

	Mean body weight (g), ±SEM				Weight decrease from previous time point (%)		
	Week 1	Week 2	Week 3	Week 4	Week 2	Week 3	Week 4
**CombiMab-2**	25.96 ± 0.49	26.33 ± 0.77	24.81 ± 0.63	23.10 ± 0.98	-1.4%	4.5%	11%
**Control**	25.22 ± 0.51	24.09 ± 0.93	21.47 ± 0.68	20.40 ± 0.95	4.7%	14.8%	18.9%
**T test**	0.299	0.071	0.006	0.051	0.072	0.006	0.094

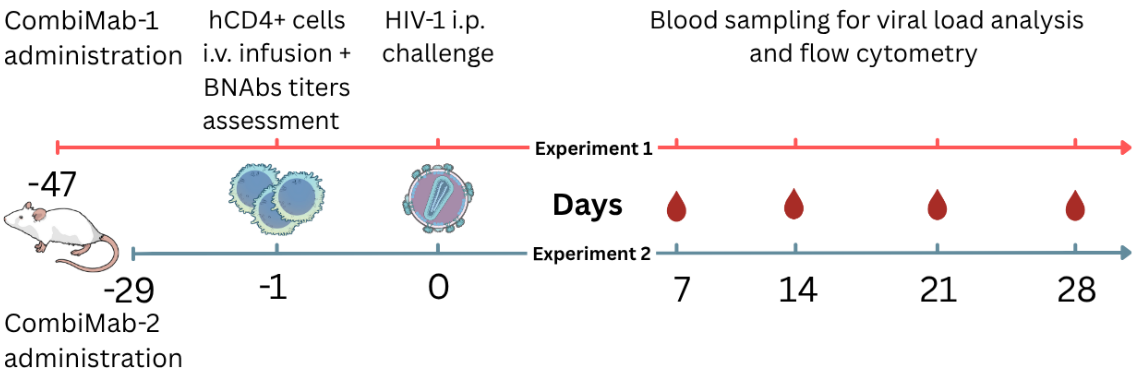

Figure S1 Experimental timelines for CombiMab-1 and CombiMab-2 studies showing the schedules of rAAV administration, HIV-1 challenge, and subsequent analyses

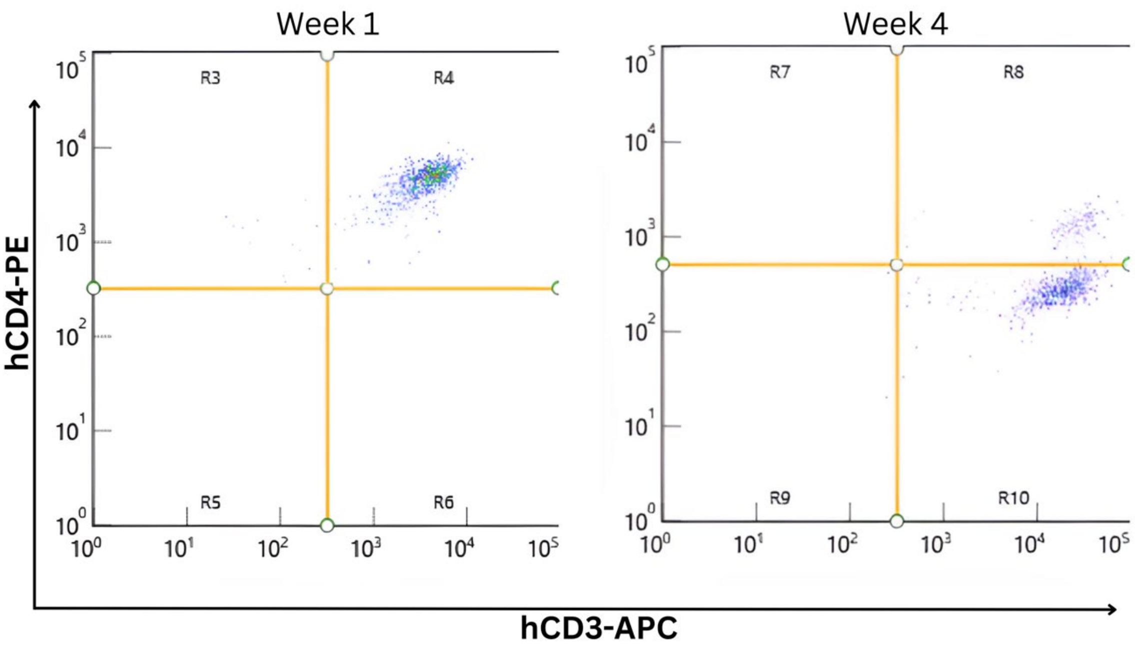

Figure S2 Flow cytometric detection of $CD3^{+}CD4^{-}$ T lymphocytes (gate R10) in mice transplanted with purified $hCD4^{+}$ cells

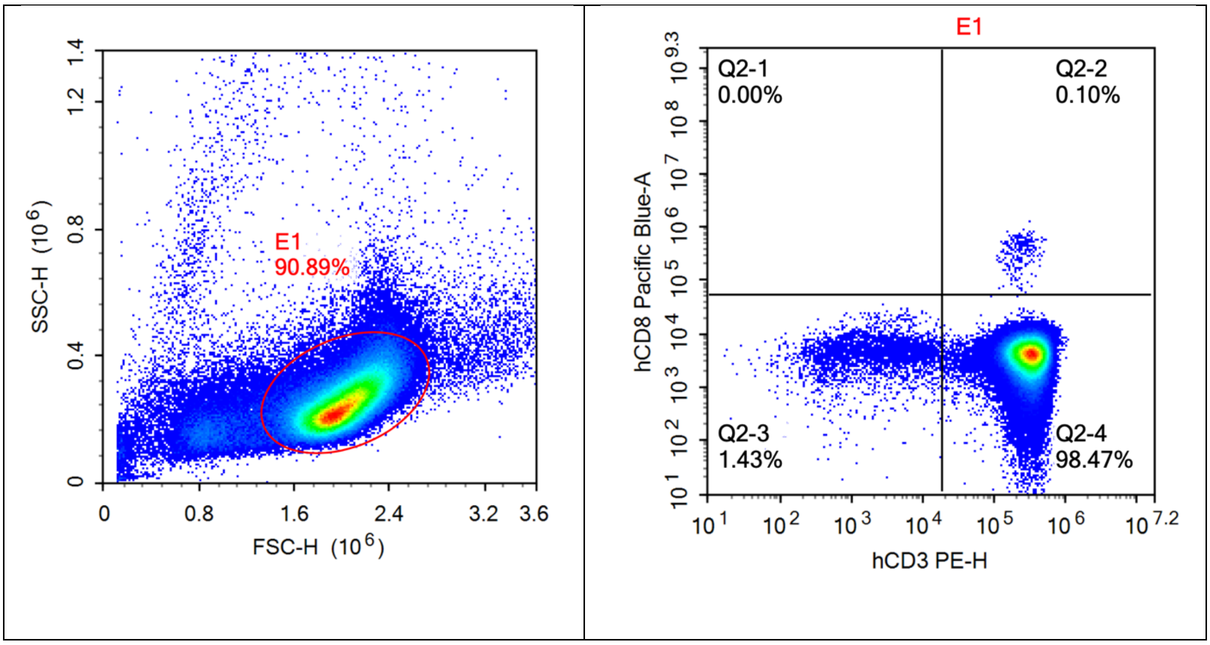

Figure S3 Flow cytometric analysis of $CD4^+$ lymphocytes after recovery from cryopreservation to assess the presence of residual $CD3^+CD8^+$ cells (gate Q2-2)
